# Design of a new lyoprotectant increasing freeze-dried *Lactobacillus* strain survival to long-term storage

**DOI:** 10.1186/s12896-021-00726-2

**Published:** 2021-11-12

**Authors:** Aurore Bodzen, Audrey Jossier, Sébastien Dupont, Pierre-Yves Mousset, Laurent Beney, Sophie Lafay, Patrick Gervais

**Affiliations:** 1 UMR Procédés Alimentaires et Microbiologiques, University Bourgogne Franche-Comté, AgroSup Dijon, PAM UMR A 02.102, 21000 Dijon, France; 2 Indigo Therapeutics, 5 rue Salneuve, 75017 Paris, France

**Keywords:** *Lactiplantibacillus*, Long-term storage, Water activity, Bound water, Lyoprotectant, Freeze-drying, Micellar casein

## Abstract

**Background:**

Stabilization of freeze-dried lactic acid bacteria during long-term storage is challenging for the food industry. Water activity of the lyophilizates is clearly related to the water availability and maintaining a low a_w_ during storage allows to increase bacteria viability. The aim of this study was to achieve a low water activity after freeze-drying and subsequently during long-term storage through the design of a lyoprotectant. Indeed, for the same water content as sucrose (commonly used lyoprotectant), water activity is lower for some components such as whey, micellar casein or inulin. We hypothesized that the addition of these components in a lyoprotectant, with a higher bound water content than sucrose would improve lactobacilli strains survival to long-term storage. Therefore, in this study, 5% whey (w/v), 5% micellar casein (w/v) or 5% inulin (w/v) were added to a 5% sucrose solution (w/v) and compared with a lyoprotectant only composed of 5% sucrose (w/v). Protective effect of the four lyoprotectants was assessed measuring *Lactiplantibacillus plantarum* CNCM I-4459 survival and water activity after freeze-drying and during 9 months storage at 25 °C.

**Results:**

The addition whey and inulin were not effective in increasing *Lactiplantibacillus plantarum* CNCM I-4459 survival to long-term-storage (4 log reduction at 9 months storage). However, the addition of micellar casein to sucrose increased drastically the protective effect of the lyoprotectant (3.6 log *i.e*. 0.4 log reduction at 9 months storage). Comparing to a lyoprotectant containing whey or inulin, a lyoprotectant containing micellar casein resulted in a lower water activity after freeze-drying and its maintenance during storage (0.13 ± 0.05).

**Conclusions:**

The addition of micellar casein to a sucrose solution, contrary to the addition of whey and inulin, resulted in a higher bacterial viability to long-term storage. Indeed, for the same water content as the others lyoprotectants, a significant lower water activity was obtained with micellar casein during storage. Probably due to high bound water content of micellar casein, less water could be available for chemical degradation reactions, responsible for bacterial damages during long-term storage. Therefore, the addition of this component to a sucrose solution could be an effective strategy for dried bacteria stabilization during long-term storage.

## Background

Preservation of lactic acid bacteria (LAB) by freeze-drying is commonly used for long-term storage of functional food supplements and starters [1]. Freeze-drying removes intracellular water from bacteria until a low water activity (a_w_) level (a_w_ ≤ 0.2) is reached, which results in the reduction or abortion of cell metabolic activities [2]. Freeze-drying process is made up of three steps: freezing which is the main critical step for maintaining bacterial viability during freeze-drying, sublimation (first drying step) and a secondary drying step. During freezing, ice first forms in the extracellular medium due to a higher solute concentration in cells than in the extracellular medium. Extracellular ice formation increases extracellular solute concentration resulting in an osmotic gradient between the cell and the extracellular medium [3]. This difference in osmotic pressure causes water outflow of the cell and cell dehydration. Cells damages occur during the freezing step depend on the kinetic of cooling. For rapid cooling rates, water is retained within the cells, leading to the formation of intracellular ice and cell damage [4, 5]. Moreover, the increase of cell surface/volume ratio resulting from rapid cooling rates causes membrane deformations such as shrinkage and vesiculation [6, 7]. Vesiculation induces membrane surface depletion and then permeabilization during rehydration because of the lack of membrane surface [8]. If a slow cooling rate is used, cellular damages are caused by hyperosmotic stress and severe dehydration [9]. Dehydration results in high solute concentration in the extracellular medium (“cryoconcentration”) and therefore in high osmotic stress. Cryoconcentration during freezing creates irreversible damages to the macromolecules, particularly proteins of cell surface, acting directly on bacteria viability [10]. Then, during the second step of freeze-drying (sublimation), pressure reduction in the freeze-drier causes the sublimation of the frozen water of the bacterial formulation [11]. The product temperature has to be maintained below the glass transition temperature of the protectant (T_g_) to remain in the amorphous glassy state throughout freeze-drying and storage and to prevent the collapse of the matrix structure [12]. Moreover, sublimation must be completed before the product temperature reaches the melting point of ice (0 °C). Therefore shelf temperature rise must be controlled to avoid product collapse or melting [13]. Following sublimation, bound water of the product is removed during a secondary drying step, which includes a slight rise in temperature. During this step, the product temperature also has to be maintained below the T_g_ of the protectant. The secondary drying is completed when low a_w_ values of the product are reached (usually below 0.2).

Regulations on functional food supplements impose their stability (*i.e*. survival of bacteria) for two years of storage usually at 25 °C. Bacterial damages due to freeze-drying (especially due to the freezing step) result in bacterial death during rehydration, highlighting the need of suitable storage conditions. Storage conditions such as a_w_, temperature, presence of light and gaseous atmosphere, are key factors in the loss of viability and generally more than 1 log of loss is observed for probiotics preparation over one month of storage at 25 °C [14]. Cell damages mechanisms are as diverse as protein aggregation, lipid oxidation or Maillard reaction of reducing sugars [13]. Most used protection strategies to prevent freeze-drying and storage cell death are the control of the cooling rate, the addition of lyoprotectants and the control of storage conditions.

Although freezing is the first step of freeze-drying, there is a large difference between cryoprotectants and lyoprotectants. Cryoprotectants are only used when bacteria are stored in frozen form. Glycerol, the most widely used cryoprotectant, is not recommended as a lyoprotectant. Indeed, the percentage of unfrozen water in glycerol is very high (about 45%) [15], thus increasing the duration of the secondary drying and the costs of freeze-drying. In addition, because pure glycerol is liquidous at storage temperature and the T_g_ of anhydrous glycerol is very low (− 93 °C) [16], maintaining the system temperature below the T_g_ during freeze-drying and storage is not possible, resulting in matrix collapse. The molecules used as lyoprotectants must therefore meet several criteria and their addition should result in (1) an initial withdrawal of intracellular water before freeze-drying, preventing damages due to freezing, (2) formation of an amorphous glass matrix with an extreme viscosity (vitrification) [17] which protects protein structure and stability [18] and increases cell stability thought freeze-drying and long-term storage [19], (3) an increase of the T_g_ of the formulation, with T_g_ as high as possible so that the product temperature is appropriate for freeze-drying and that the product remains in a glassy state during storage despite generally high temperatures (25 °C) [12, 20, 21], iv) hydrogen bonds formation with membrane proteins (water replacement hypothesis), thus preserving the native structure of membrane proteins during the withdrawal of water [22, 23]. Non-reducing disaccharides, such as sucrose are generally used as lyoprotectant.

Numerous studies have shown that maintaining a low relative humidity (RH) of the atmosphere i.e. a low a_w_ of the product during storage (dried product equilibrated at different RH using saturated salt solution before or during storage) increases bacterial stability over long-term storage [21, 24–32]. When the bacteria formulation moisture content increases during storage, the “dormant state” of the bacteria is reverted and cell damages (lipid oxidation, protein denaturation) can occur often resulting in cell death [19]. In addition, a low a_w_ during storage could also be obtained using molecules with a high bound water content. Indeed, as observed by comparison of sorption isotherms, at similar water content, a lower a_w_ is achieved with these components. Among these components, whey, micellar casein or inulin are highly relevant in this context.

Skim milk was widely investigated as a lyoprotectant to maintain bacterial viability during long-term storage [33, 34]. Skim milk is composed of 50% (w/w) lactose (reducing disaccharide) and 30% (w/w) milk proteins (e.g., whey, casein). Comparing sorption isotherms of these milk proteins [35–37] with sucrose [38] leads us to the conclusion that these proteins have a higher bound water content than sucrose (micellar casein > whey > sucrose). Whey and micellar casein can also increase the T_g_ of a formulation due to their very high T_g_ (T_g_ anhydrous whey: 127 °C; T_g_ anhydrous casein: 132 °C) compared to sucrose (T_g_ anhydrous sucrose: 77 °C) [39]. Fructo-oligosaccharides (FOS), particularly inulin, have also been used to increase LAB survival to long-term storage [40, 41]. However, these studies did not monitor a_w_ during storage [40] or did not use the freeze-drying method to stabilize bacteria [41]. Inulin, a well-recognized prebiotic, is an heterogeneous mixture of oligosaccharides resulting in a high T_g_ (126 °C for DP10) [39] which maintains more easily the glass state during storage than disaccharides. The comparison of inulin sorption isotherm [42, 43] with sucrose ‘s [39], shows that inulin also has a higher bound water content than sucrose. Moreover, the benefits of the conjoint use in a lyoprotectant of molecules interacting with membrane, such as disaccharides and polysaccharides with high T_g_, to prevent bacterial death during freeze-drying and long-term storage have already been reported [44].

In this context, a lyoprotectant composed of sucrose and a component with a high bound water content seemed to be a suitable strategy to protect bacteria from freeze-drying and also long-term storage damages. In this study, we evaluated the effect of a lyoprotectant containing sucrose + whey, sucrose + micellar casein or sucrose + inulin in comparison with a lyoprotectant only composed of sucrose, to increase LAB strains survival to a freeze-drying process and a long-term storage of 9 months at 25 °C. Sucrose, the most used lyoprotectant for lactic acid bacteria, was chosen because it forms an amorphous matrix, hydrogen bonds with the membrane proteins and its addition leads to a first outflow of water from the cells (less water outflow during freezing). whey, micellar casein and inulin were chosen for their similar T_g_ but different bound water content (micellar casein > inulin > whey > sucrose).

## Results

### Bacterial survival to freeze-drying

**Fig. 1:**
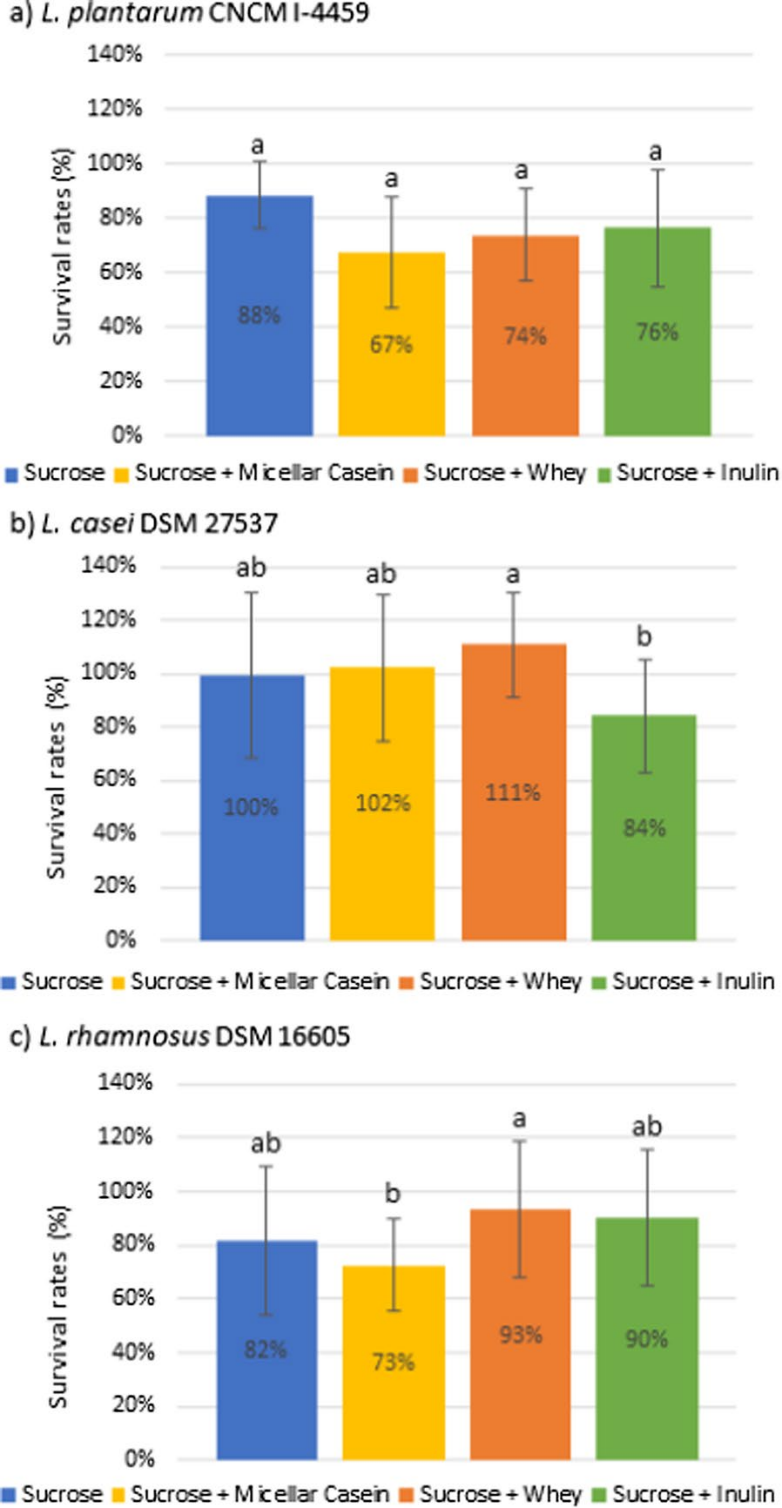
Survival rates to freeze-drying (%) of *L. plantarum* CNCM I-4459 (**a**), *L. casei* DSM 27537 (**b**) et *L. rhamnosus* DSM 16605 (**c**) with different lyoprotectants. Values represent mean ± standard deviation obtained from independent triplicates. Different letters indicate significant differences between lyoprotectants (Tukey’s Honest Significant Difference test, *p* < 0.05)

The survival rates of the three LAB strains (*L. plantarum* CNCM I-4459, *L. casei* DSM 27537 and *L. rhamnosus* DSM 16605) to freeze-drying process were determined using four different lyoprotectants and are presented in Fig. 1.

No significant difference in survival rates of *L. plantarum* CNCM I-4459 (Fig. 1a) was observed between the four lyoprotectants (*p* > 0.05) whereas the survival rates of *L. casei* DSM 27537 (Fig. 1b) and *L. rhamnosus* DSM 16605 (Fig. 1c) were significantly different. The lyoprotectant containing inulin was the least efficient to protect *L. casei* DSM 27537 to freeze-drying and the addition of micellar casein or whey did not improve the protection of sucrose. The survival rates of *L. rhamnosus* DSM 16605 were significantly higher with the lyoprotectant containing whey than with the lyoprotectant containing micellar casein (*p* < 0.05). In addition, *L. rhamnosus* DSM 16605 survival rates to freeze-drying with the lyoprotectants containing whey and inulin were not significantly different from survival rates with the lyoprotectant containing only sucrose (*p* > 0.05).

A one-sample t-test has also allowed to show that the freeze-drying survival percentages of the three strains with the lyoprotectant containing only sucrose were not significantly different from 100% (*p* > 0.05).

### Bacterial survival to long-term storage

At each storage time (0 month, 0.5 month, 1 month, 3 months and 9 months), *L. plantarum* CNCM I-4459 cultivable biomass (CFU/mL) was enumerated. As same as previous results (Fig. 1), there was no significant difference between cultivable bacteria obtained after freeze-drying (0) between the four lyoprotectants.

**Fig. 2:**
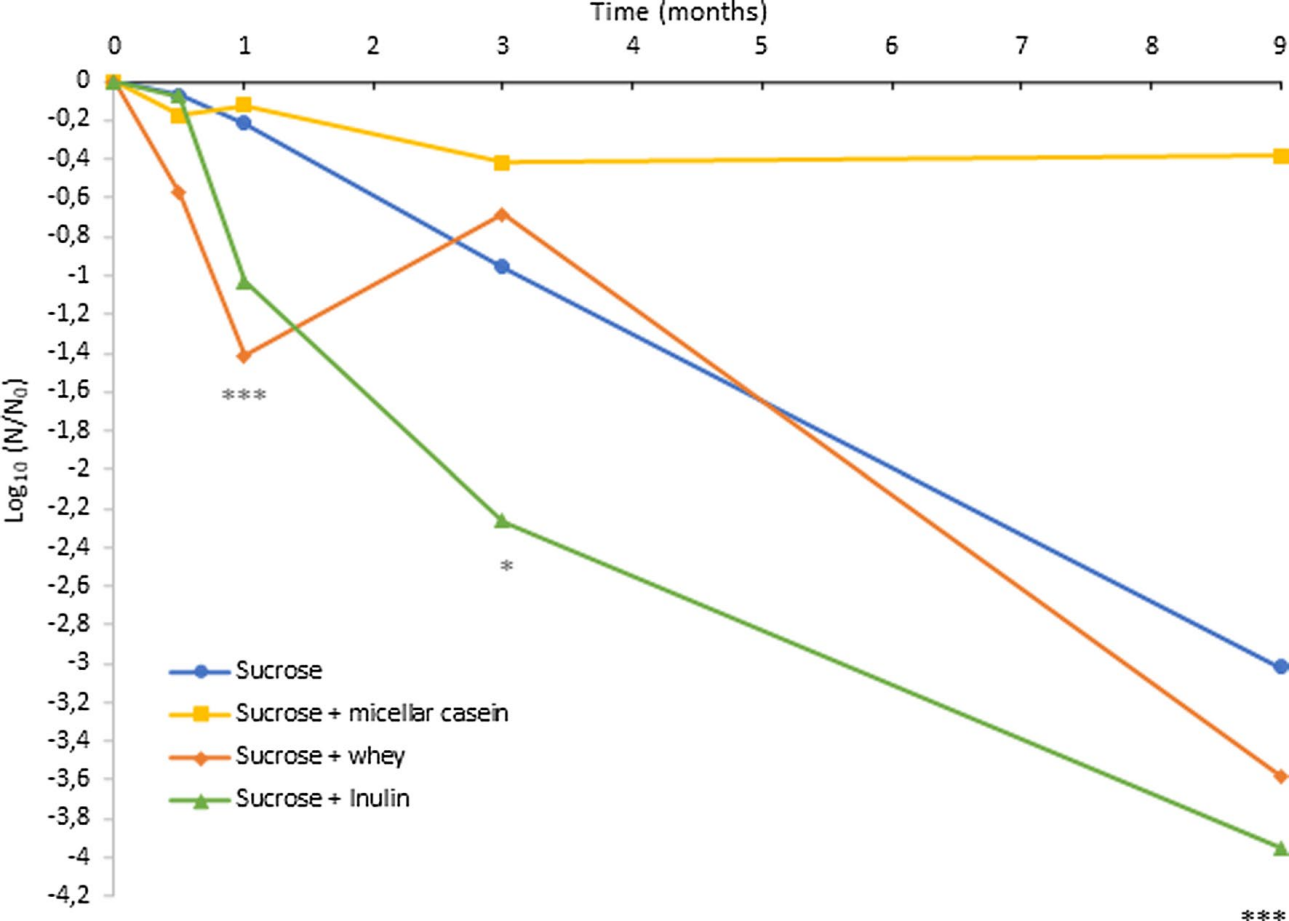
Logarithmic reduction of *L. plantarum* CNCM I-4459 cells during long-term depending on the lyoprotectant. Values represent mean ± standard deviation obtained from independent triplicates. The asterisks indicate a significant difference between lyoprotectants (*: *p* < 0.05; **: *p* < 0.01; ***: *p* < 0.001, ANOVA)

The loss of bacterial cultivability at each storage time and depending on the lyoprotectant, expressed as log_10_ (N/N_0_), is presented in Fig. 2.

Bacterial cultivability at each storage time and depending the four lyoprotectants is presented in Table 1.

**Table 1:** Cultivable biomass of L. plantarum CNCM I-4459 (CFU/mL) for each storage time depending on the lyoprotectant

Storage time	Lyoprotectants							
	Sucrose		Sucrose + Micellar Casein		Sucrose + Whey		Sucrose + Inulin	
0 month	1.9 × 10^10^	a	1.4 × 10^10^	a	1.8 × 10^10^	a	1.8 × 10^10^	a
	± 2.5 × 10^9^		± 1.9 × 10^9^		± 1.2 × 10^9^		± 1.8 × 10^9^	
0.5 month	1.6 × 10^10^	a	9.4 × 10^9^	ab	4.8 × 10^9^	b	1.1 × 10^10^	ab
	± 2.0 × 10^9^		± 2.1 × 10^9^		± 9.7 × 10^8^		± 6.6 × 10^9^	
1 month	1.1 × 10^10^	a	1.0 × 10^10^	a	7.9 × 10^8^	b	1.6 × 10^9^	b
	± 4.6 × 10^9^		± 2.0 × 10^9^		± 7.7 × 10^8^		± 6.4 × 10^8^	
3 months	1.5 × 10^9^	ab	3.4 × 10^9^	a	2.6 × 10^9^	a	1.0 × 10^8^	b
	± 1.0 × 10^8^		± 3.0 × 10^9^		± 3.6 × 10^9^		± 1.4 × 10^8^	
9 months	1.9 × 10^7^	b	5.8 × 10^9^	a	4.7 × 10^6^	b	1.9 × 10^6^	b
	± 3.2 × 10^6^		± 9.9 × 10^8^		± 1.0 × 10^6^		± 1.0 × 10^6^	

Values represent mean ± standard deviation obtained from independent triplicates. Different letters indicate significant differences between lyoprotectants (Tukey’s Honest Significant Difference test, *p* < 0.05)

Firstly, at 15 days, cultivable bacteria were similar between lyoprotectants containing micellar casein, whey and inulin (Table 1), however the loss of cultivability (Fig. 2) was higher for the lyoprotectant containing whey (0.6 log) than with the lyoprotectants containing micellar casein (0.2 log) and inulin (0.1 log). At 1 month, cultivable bacteria obtained with the lyoprotectant containing micellar casein was significantly higher than cultivable bacteria obtained with the lyoprotectants containing whey and inulin (Table 1). The lyoprotectant containing micellar casein was more efficient to protect bacterial survival (0.1 log reduction) than the lyoprotectants containing whey (1.4 log reduction) or inulin (1 log reduction) (Fig. 2). Moreover, cultivable bacteria obtained with the lyoprotectant containing micellar casein was not significantly different between 0,5 month and 1 month. At 3 months and 9 months, the loss of cultivability remained low for the lyoprotectant containing micellar casein (0.4 log reduction) (Fig. 2). With the lyoprotectant containing only sucrose and the lyoprotectants containing whey and inulin, the loss of cultivability at 9 months storage was between 3 to 4 log (Fig. 2). Moreover, at 9 months, cultivable bacteria obtained with the lyoprotectant containing micellar casein was significantly higher than cultivable bacteria obtained with the lyoprotectant containing only sucrose and the two others lyoprotectants (*p* < 0.05, ANOVA) (Table 1).

### Water activity measurements during long-term storage

**Fig. 3:**
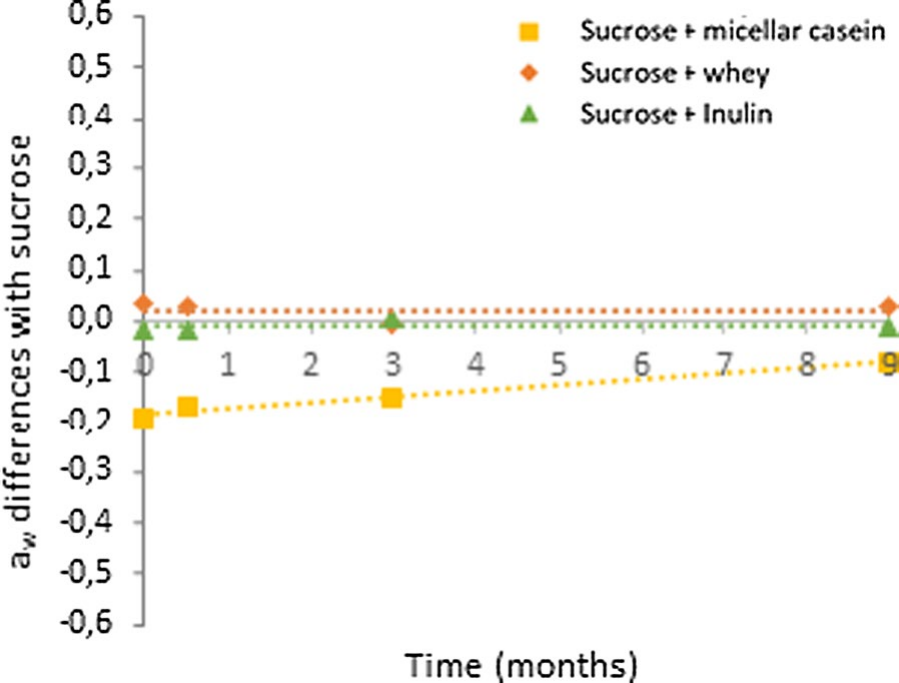
Differences between water activity of a lyoprotectant containing only sucrose and water activity of others lyoprotectants for *L. plantarum* CNCM I-4459 over storage time. Linear regression models are represented by dotted lines

Throughout storage, sucrose a_w_ values range from 0.247 to 0.291. Concerning a_w_ values after freeze-drying (at 0 month), there were differences between the four lyoprotectants. Indeed, for sucrose, sucrose + whey and sucrose + inulin, a_w_ were in the range 0.229 and 0.279 whereas a_w_ was much lower for sucrose + micellar casein and equal to 0,053. A slight increase in a_w_ values was observed for all the others lyoprotectants mainly due to water sorption during the a_w_ measurements. Therefore, to remove the effect of RH variations of the osmometer room between the different storage times, the results were expressed through a_w_ differences. Differences between a_w_ of the lyoprotectant containing only sucrose and a_w_ of lyoprotectants containing micellar casein, whey or inulin are presented in Fig. 3.

Differences between a_w_ of the lyoprotectant containing only sucrose and a_w_ of lyoprotectants containing whey or inulin were close to 0 throughout storage as shown by the linear regression lines. Consequently, a_w_ values were almost stable during storage for these lyoprotectants. Therefore, the sealing of the samples was effective to limit a_w_ variations during storage. Regarding the lyoprotectant containing micellar casein, although a slight increase was observed, a_w_ values can also be considered constant over time. Moreover, for the lyoprotectant containing micellar casein, a_w_ values (mean a_w_ value: 0.13 ± 0.05) were significantly lower (*p* < 0.05, ANOVA) than for the others lyoprotectants. Indeed, means a_w_ values with sucrose, sucrose + whey and sucrose + inulin were 0.26 ± 0.02, 0.29 ± 0.03 and 0.26 ± 0.02, respectively.

## Discussion

In this study, sucrose was an effective lyoprotectant allowing to preserve bacterial viability during a freeze-drying process, which is consistent with previous reports found in literature [20, 45, 46]. Non-reducing disaccharides are effective lyoprotectants for bacterial freeze-drying [20], including lactic acid bacteria freeze-drying [45, 46]. The main reason of this protection is the water exit from the cell provoked by this disaccharides addition which affords a good resistance to freezing preventing from membrane vesiculation [7]. Moreover several studies have also demonstrated that sucrose can form an amorphous glass and hydrogen bonds with membrane proteins thus preventing cell damage from freeze-drying and subsequent storage [21, 47]. In our work the association of micellar casein, whey or inulin to sucrose had no significant effect on bacterial survival to freeze-drying. However, because of survival rates with sucrose were already close to 100%, the effect of the addition of micellar casein, whey or inulin to sucrose on survival rates to freeze-drying of the strains can hardly be seen.

The results of this study also show that there is a significant influence of the lyoprotectant on *L. plantarum* CNCM I-4459 long-term storage survival. Contrary to what has been expected, the addition of whey or inulin to a sucrose solution did not increase the survival of this strain to long-term storage. However, the lyoprotectant containing micellar casein was significantly better for stabilizing the cultivable bacterial biomass during long term storage. Despite an initial mortality during the first three months of storage, the number of cultivable bacteria was then stable up to 9 months of storage resulting in 0.4 log reduction. The mortality observed during the first three months of storage can be explained by the fact that the cells which have been damaged by freeze-drying, also undergo damage throughout storage (lipid oxidation, protein aggregation). Subsequently, freeze-dried cultivable bacterial biomass with sucrose + micellar casein remained stable if these cells had not been previously damaged by freeze-drying. A 1-log reduction achieved after 12 months of storage has been identified as economically viable [14]. Therefore, the lyoprotectant containing micellar casein is an effective and economically acceptable lyoprotectant for stabilizing bacterial biomass during freeze-drying and long-term storage.

**Fig. 4:**
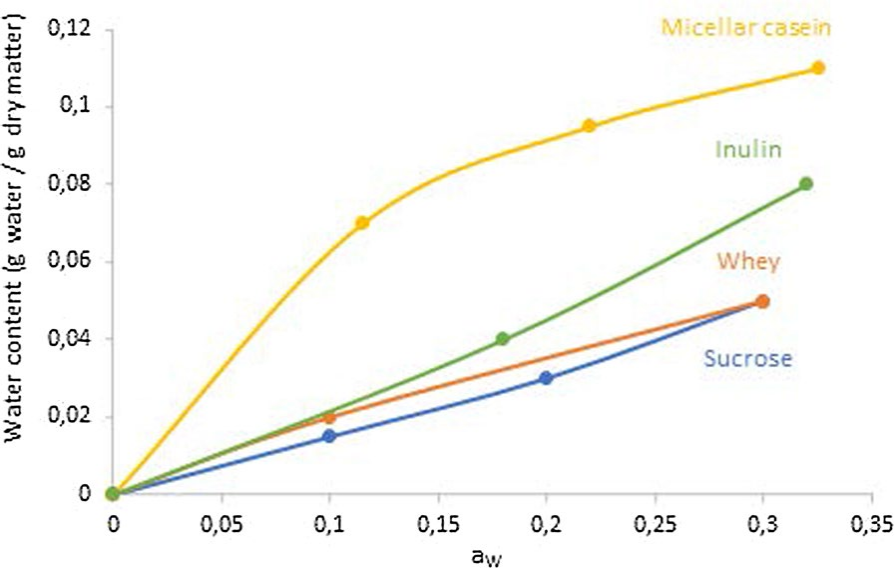
Sorption isotherms of sucrose at 25 °C (from [38]), of freeze-dried micellar casein at 27 °C (from |35]), of whey protein concentrate (from [37]) and of inulin at 23 °C (from [43])

Our results indicate that *L. plantarum* CNCM I-4459 storage survival depends on the a_w_ after freeze-drying and its maintenance during storage. Hence, there was a clear link between the a_w_ of the product and long-term storage survival, since the lower the a_w_, the higher the bacterial survival to storage. The difference in a_w_ after freeze-drying between the lyoprotectants with micellar casein and the other lyoprotectants can be explained through the sorption isotherms of these components presented in Fig. 4.

Indeed, sorption isotherm of micellar casein is very different from sorption isotherms of sucrose, whey or inulin [35–38, 42, 43]. Considering that the water content of both samples was close after freeze-drying (around 0.04 g water/g dry matter as previously obtained in our laboratory (data not shown)), then the corresponding a_w_ values obtained from these isotherms in Fig. 4 coincide with the a_w_ values measured after freeze-drying of the lyoprotectants. The difference between a_w_ values of the lyoprotectant containing only sucrose and the lyoprotectant containing micellar casein is very high after freeze-drying for the same water content, which had a significant impact during storage and in particular in the acceleration of chemical degradation. Indeed, below the T_g_, chemical degradations reactions such as Maillard reaction, proteins aggregations, deamidation and lipid oxidation of membrane fatty acid increase with a_w_ [48, 49]. A few studies have also shown higher bacterial inactivation with higher a_w_ during long-term storage and, have linked an increase of previously mentioned chemical reactions to bacterial death [25, 27, 32]. Therefore, a lower a_w_ during storage must have limited biological reactions, thus increasing bacterial stability during storage.

To understand the particular sorption properties of micellar casein, we examined properties of casein-based dairy powders. Casein-based dairy powders are currently used in food industry in cheese and yogurt manufacturing or in nutritional preparations due to their high protein content [50]. Caseins powders are generally dried by spray drying and the removal of water leads to the formation of aggregates of interlinked casein micelles. An increase of interactions between and within micellar casein was also noticed during storage of milk protein concentrate powders [51]. Moreover, the formation of a crust on the surface of micellar casein during storage, composed of layers of fused caseins, has been observed [52]. Therefore, micellar casein structure and the layer of fused caseins on the micelles surface could explain the particular sorption properties of this protein.

Our study demonstrated that bacterial survival to long-term storage depended on the high bound water content of components used as lyoprotectants. Therefore, for lactic acid bacteria strains survival to freeze-drying and to long-term storage, a lyoprotectant containing sucrose (for freezing protection and high T_g_) and micellar casein (for high bound water content and high T_g_) could interestingly be chosen.

## Conclusion

The results of this study demonstrate the importance of the choice of the lyoprotectant for bacterial survival to long-term storage. For the same water content as for sucrose, whey and inulin, a significant lower water activity was obtained with the lyoprotectant containing micellar casein during storage. Therefore, with the lyoprotectant containing micellar casein, a higher bacterial viability to long-term storage was found. The higher bound water content of micellar casein could have resulted in less water available for chemical degradation reactions, responsible for bacterial damages during long-term storage. Therefore, the addition of micellar casein (for long term storage protection) to a sucrose solution (for freezing protection) can protect a lactic acid bacteria strain from freeze-drying stresses and from long-term storage degradation. Use of a lyoprotectant mixture with a high bound water content is thus a promising strategy for the food industry to improve lactic acid bacteria survival to long-term storage.

## Materials and methods

### Bacterial strains and stock solutions

*Lactiplantibacillus plantarum* CNCM I-4459 was provided by Novanat (Shanghai NOVANAT Co., China) and *Lacticaseibacillus casei* DSM 27537 and *Lacticaseibacillus rhamnosus* DSM 16605 strains were provided by Probiotical (PROBIOTICAL S.p.A, Italy). The three strains were cultured in MRS broth medium (*Lactobacillus* Broth acc. to De Man, Rogosa and Sharpe, Sigma-Aldrich) (pH 6.2 ± 0.2 at 25° C) at 37 °C for 24 h. The MRS medium was prepared according to the manufacturer's instructions and sterilized at 121 °C for 20 min after the addition of 0.1% (v/v) Tween 80 (Sigma-Aldrich). Cultures were then diluted to 20% in sterile glycerol (Honeywell, USA) (v/v), then aliquoted in 1 mL cryotubes and stored at -80 °C until further use.

### Culture conditions

Bacteria were streaked out (100 µl) on MRS agar and incubated 24 h at 37 °C. Following incubation, one colony was inoculated in 10 mL of MRS broth (pre-cultures). Finally, after 24 h at 37 °C, fresh MRS (10 mL) was inoculated at 1% (v/v) with pre-cultures and incubated at 37 °C.

### Freeze-drying

Four different formulas of lyoprotectants were used: sucrose, sucrose + whey, sucrose + micellar casein and sucrose + inulin. These lyoprotectants were composed of 5% (m/v) sucrose (Sigma-Aldrich) in PBS (Phosphate Buffered Saline, Sigma-Aldrich) and of 5% whey protein isolate (degree of hydrolysis = 25%, 25S, Ingrediat, France) or 5% micellar casein (87B Fluid, Ingredia, France) or 5% inulin DP10 (Fibruline® Instant, Cosucra). Bacterial cells were centrifuged (4,000 g—10 min, Eppendorf 5810 R) and pellets resuspended (10 times concentrated) in the different lyoprotectants. One milliliter of each mixture was poured into vials (amber glass vials of 5 mL) and frozen at − 80 °C (at a rate of -2 °C/min) before being freeze-dried for 24 h (FreeZone 18-Liter Console Freeze Dry System with Stoppering Tray Dryer, Purge Valve and PTFE-Coated Collector, Labconco, Kansas City, USA). Sublimation was carried out by maintaining the samples for 2 h at -40 °C (condenser temperature = − 55 °C and chamber pressure = 10 Pa), then increasing the chamber temperature to 0 °C at a speed of 0.04 °C/min. After 17 h, the chamber temperature was increased to 25 °C at a heating rate of 0.08 °C/min to carry out the secondary desorption.

### Long-term storage

Due to the large number of samples (160 for one strain) needed to measure the cultivable bacteria and the a_w_ throughout long-term storage, the influence of the four lyoprotectants on bacterial survival to long-term storage has only been studied for the strain *L. plantarum* CNCM I-4459. Moreover the strain *L. plantarum* CNCM I-4459 was selected for the long-term storage study because the freeze-drying survival and the cultivable biomass after freeze-drying (at 0 month) were not significantly different between the four lyoprotectants. After freeze-drying, vials were sealed with butyl rubble stoppers and aluminum screw caps under vacuum (10 Pa) which afforded to keep a constant a_w_ value during storage. Samples were then stored for 9 months at 25 °C in the dark. Before water activity measurements or bacterial enumeration at each storage time, the maintenance of the samples under vacuum was checked.

### Water activity measurements

Water activity of the lyophilizates was measured at 25 °C using an Aqualab CX-2 Osmometer (Decagon Devices, Pullman, WA, USA) after freeze-drying and at each storage time (15 days, 3 months and 9 months). At 1 month of storage, a_w_ has not been measured. The freeze-dried samples contained in five vials were regrouped for one a_w_ measurement, corresponding to one storage time. To remove the effect of RH variations of the osmometer room between the different storage times, the results were expressed through a_w_ differences, *i.e.* for each storage time the difference between a_w_ of sucrose and a_w_ of others lyoprotectants were calculated.

### Bacterial survival to freeze-drying and long-term storage

Cultivable bacteria were enumerated using the method of Colony Forming Units (CFU/mL and CFU/g of lyophilizate) after freeze-drying and at each storage time (15 days, 1 month, 3 months and 9 months). Rehydration was performed with 1 mL of MRS broth at 37° C to enumerate viable cells. Bacterial survival rate to the freeze-drying process (%) was expressed as the ratio between the cultivable bacteria after freeze-drying (CFU/mL) and before freeze-drying (CFU/mL). Due to the large variations between the cultivable bacteria during storage, the loss of cultivability during long-term storage was expressed in log_10_ (N/N_0_), where N corresponds to the cultivable bacteria at the time of storage (CFU/g of lyophilizate) and N_0_ corresponds to the cultivable bacteria at 0 month (CFU/g of lyophilizate).

### Statistical analysis

An analysis of variance (ANOVA) was used to investigate effects of the four lyoprotectants on freeze-drying and storage survival (*p* < 0.05). For each lyoprotectant, an ANOVA was also performed to compare cultivable bacteria and survival rates between each storage time. When significant differences were observed, Tukey’s Honest Significant Difference test was performed. Significant differences between the bacterial concentrations and survival rates were shown with different letters in the Figures. One-sample t-test was used to compare mean values with specified values (*p* < 0.05). All experiments were carried out in completely independent triplicates (n = 3), except for a_w_ measurements. The R Software v.3.3.2 (R Development Core Team, 2008) was used to statistically analyze data.

## Data Availability

The datasets used and/or analyzed during the current study are available from the corresponding author on reasonable request.

## Abbreviations

ANOVA: Analysis of variance
a_w_: Water activity
CFU: Colony forming units
LAB: Lactic Acid Bacteria
MRS: *Lactobacillus* Broth acc. to De Man, Rogosa and Sharpe, Sigma-Aldrich
RH: Relative Humidity
T_g_: Glass transition temperature of the protectant
